# Weakly Supervised Video Anomaly Detection Based on 3D Convolution and LSTM

**DOI:** 10.3390/s21227508

**Published:** 2021-11-12

**Authors:** Zhen Ma, José J. M. Machado, João Manuel R. S. Tavares

**Affiliations:** 1 Faculdade de Engenharia, Universidade do Porto, Rua Dr. Roberto Frias s/n, 4200-465 Porto, Portugal; zhen.ma@fe.up.pt; 2 Departamento de Engenharia Mecânica, Faculdade de Engenharia, Universidade do Porto, Rua Dr. Roberto Frias s/n, 4200-465 Porto, Portugal; jjmm@fe.up.pt

**Keywords:** video anomaly detection, three-dimensional convolution, LSTM, weakly supervised, spatial-temporal features, max-pooling

## Abstract

Weakly supervised video anomaly detection is a recent focus of computer vision research thanks to the availability of large-scale weakly supervised video datasets. However, most existing research works are limited to the frame-level classification with emphasis on finding the presence of specific objects or activities. In this article, a new neural network architecture is proposed to efficiently extract the prominent features for detecting whether a video contains anomalies. A video is treated as an integral input and the detection follows the procedure of video-label assignment. The extraction of spatial and temporal features is carried out by three-dimensional convolutions, and then their relationship is further modeled using an LSTM network. The concise structure of the proposed method enables high computational efficiency, and extensive experiments demonstrate its effectiveness.

## 1. Introduction

In the past few decades, video anomaly detection has increasingly become a research focus because of its wide application, such as in public safety and online video censorship. Along with the popularity of camera hardware, the number of videos acquired by smartphones and surveillance cameras has increased so drastically, that manual processing of these videos becomes unfeasible in many scenarios due to its low efficiency. 

Anomaly detection refers to the problem of finding irregular patterns that do not conform to the expectation [1]. Anomalies in a video include not only common irregularities, like vandalism, assault, and traffic accidents, but also some events under certain contexts such as a car entering a pedestrian-only zone. Though it seems that the identification of an abnormal object or event is the unique critical factor to consider, the context of a video is of equal importance for detection. Accordingly, video anomaly detection is different from human action recognition and event recognition, because it is more complicated to define a video anomaly than to barely detect an event or action; it involves a much wider range of activities, and can have a large inter-class variance as a result of complex contexts. In addition, given that video anomaly detection focuses on whether a video contains anomalies, it is as well different from video anomaly localization, which aims to identify all the abnormal frames in a video. The recent research trend suggests that detection and localization can be combined into a single end-to-end pipeline; however, the performance of such a combination remains to be explored since using a video anomaly detector to find the localization of abnormal frames may not be accurate as expected due to its nonlinear characteristics.

In practice, anomaly usually happens in a short time slot, while the principal part of the video can still be considered normal. A natural way to find out whether a video contains an anomaly is to follow the idea of multiple instance learning (MIL), where a video is considered normal only when there is no anomaly in all its segments. To comply with this requirement, it is necessary to identify both the activities and the context of a video. In the early works towards video anomaly detection, features such as object trajectory and spatiotemporal context were manually selected and calculated for classification [2,3]. Scenarios considered in these works were simple: videos were assumed to be well-trimmed, and each frame was required to be annotated. However, it is very time-consuming to obtain such a video dataset; for that reason, no large-scale datasets were available for training and objectively evaluating an algorithm or framework. In recent years, deep learning methods have become the dominant technique in computer vision and pattern recognition. Unlike the traditional methods, features used in common deep learning methods are selected and optimized automatically. Deep learning methods achieved impressive performances in video anomaly detection and have improved benchmarks on many datasets by a large margin [4], and their applications have been proved to be effective especially in complicated scenarios. 

Based on the way of human intervention and the information used for training, usual deep learning methods for video anomaly detection can be classified into supervised, unsupervised, and weakly supervised methods. Supervised deep learning methods rely on the clear identifications of normal and abnormal activities; successful training depends on sufficient samples with accurate frame-level annotations, so their applications are limited by the availability of large-scale datasets. Unsupervised methods, on the other hand, do not require labeled samples; instead, the training set is composed of only normal videos to understand how the normal scenarios should be. Subsequently, a video is considered abnormal if it is dissimilar to the normal ones in the training set; the robustness of a model, therefore, relies on how representative the training videos are, and how the difference between normal and abnormal videos is defined and measured. Insufficient training or unrepresentative training samples can lead to a high false-positive rate. 

**Figure 1:**
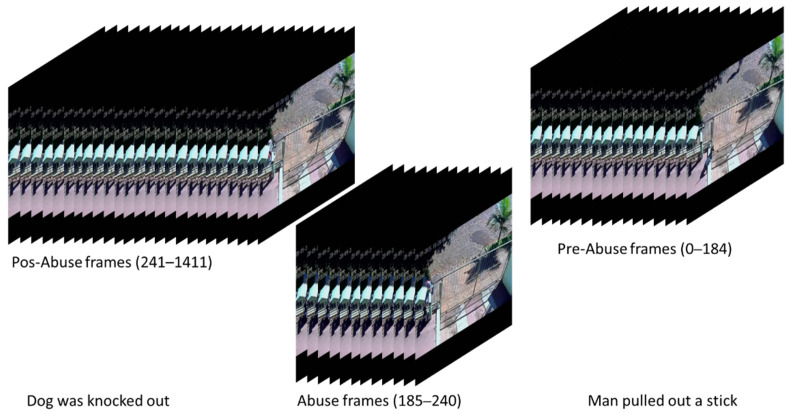
An example of a weakly surprised anomaly video in the UCF-Crime dataset [5] under the subcategory of abuse (Abuse028_x264.mp4). (The video contains 1412 frames, where the abuse scene lasted around 56 frames corresponding to approximately 4% of the total frames).

Many powerful and effective deep learning methods have been proposed [4]; along with improved accuracy, the architectures of neural networks become deeper, and therefore, more training samples are required to avoid underfitting and biased training. Since assigning a unique label to a video is much easier than annotating each frame, the recently published weakly supervised datasets provide a good solution for this problem [5,6,7]. An example of these videos is illustrated in Figure 1. These large-scale datasets contain much more training samples than other available datasets. Meanwhile, these video-level labels provide important guidance for a network to discern anomalies from normal scenes. With the emerging of tremendous videos generated every day, assigning a video-level annotation turns to be more reasonable for video anomaly detection. Consequently, methods for weakly supervised datasets have become popular. 

Though a deep learning method requires no manual feature engineering, defining an effective architecture and choosing a suitable framework are critical for its performance. The focus of video anomaly detection is to identify events, activities, and contexts presented in temporal-sequential frames; therefore, dynamic spatial–temporal features are required. In this article, a new framework is proposed to efficiently detect anomalies using weakly supervised video datasets. Three-dimensional (3D) convolutions with max-pooling are adopted to extract the prominent spatial–temporal information, and then the long short-term memory (LSTM) network [8] is used to further model the relationship between these features for classification. 

## 2. Materials and Methods

### 2.1. Related Work

Many deep learning methods have been proposed for video anomaly detection. The main difference between these existing methods is the way of how to discern the anomalies from the normal scenarios. A video or a video frame is commonly handled as an outlier when an object or event presented in it is significantly different from the ones learned from the training set. Autoencoder is a commonly adopted technique for these methods; with sufficient samples, the trained autoencoder can generate small reconstruction errors for normal videos and large errors for abnormal ones. In [9], a fully convolutional network (FCN) was proposed to learn both motion features and regular patterns; the regularity score of a video is computed based on the reconstruction error of the autoencoder. The LSTM architecture was later used in [10] to model the temporal relationship between video frames; the combination of FCN and LSTM achieved better performances. Other efficient networks, like recurrent neural network (RNN) and inception models, were also integrated into the autoencoder methods in [11,12], which further improved the performance of detection. 

Some of the methods used transfer learning technique and combined the extracted features with other classification methods to carry out the detection [7,13,14,15,16]; for example, in [14,15], with the features extracted from the pre-trained VGG model [17], unmasking processes were carried out for assigning anomaly scores of video frames. Anomaly detection was treated as multi-class classification in [16], and then methods such as k-means clustering and support vector machine were used for classification. Recently, generative adversarial networks (GANs) were also proposed for anomaly detection in [18,19]; a properly trained generator can produce highly realistic fake frames that are indistinguishable for the discriminator, then a high anomaly score will be assigned to a video when a sequential video frame is significantly different from the predicted one.

Along with the availability of large-scale weakly classified datasets, weakly supervised methods are the recent focuses in video anomaly detection. For example, the methods proposed in [5,20,21,22] adopted the ranking frameworks for the detection, and in order to capture the anomalies, each video in the training set was divided into 32 video segments that were fed separately into the network for training; the outputs of these video segments, which corresponding to their scores, were then ranked and the highest score was chosen to indicate whether the input video contains anomalies. These weakly supervised methods achieved impressive performance. Nevertheless, even with the small segments of a video, a large score can be assigned to a normal scene and a low score for an abnormal scene. Therefore, training with video segments can still lead to bias of discerning anomalies from normal ones. A graph convolution neural (GCN) network was proposed to solve this problem in [23]; the wrongly selected normal segment in an anomaly video was treated as a label noise and an iterative optimization process was used to eliminate label noise. The proposed GCN network achieved better results; however, the training was computationally expensive and can lead to unstable performance due to unconstrained latent space.

### 2.2. Proposed Method

Most of the current weakly supervised models adopted the way of dividing a video into a predefined number of segments to identify the segments that contain anomalies, like the way for a strongly supervised dataset. However, such a division is not accurate in most cases because without knowing the exact spatial and temporal location of anomalies, the integrity of an event can be broken or inter-related events can be separated, which will confuse the classifier; meanwhile, handling multiple segments is time-consuming, and class imbalance may become a problem during the training given anomaly only happens in a small time slot.

**Figure 2:**
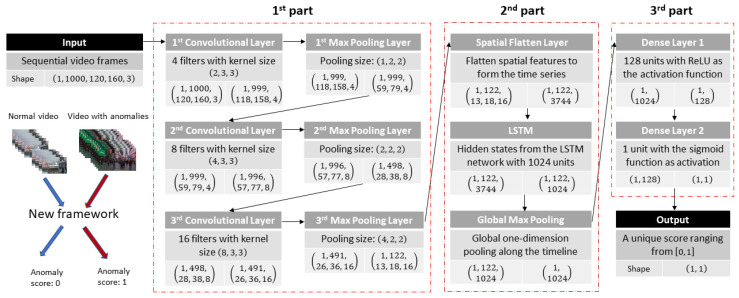
The architecture of the proposed framework. The new framework consists of three parts, each one delineated by a red rectangle: the first one includes three blocks composed of one convolutional layer followed by a pooling layer; the second one is composed of an LSTM network and a global pooling layer, and the third one is a combination of two dense layers to generate the final score. The changes of input and output shapes are illustrated by a video assumed to have 1000 frames of 3 color channels with height and width of 120 and 160 pixels, respectively. The framework does not have restrictions on image size, nor the number of frames; it takes a video as an integral input and outputs a unique score to indicate whether it contains anomalies.

To solve the aforementioned problems, a more natural way was adopted to decide whether a video contains anomalies conforming to the idea of MIL: a video is treated as an integral input and is considered normal only when it contains no anomaly. Max-pooling operations are used to replace the division of a video and to capture the most prominent spatial-temporal features corresponding to possible anomalies. In such a way, a video is classified based on the unique score generated by the framework. Hence, detection of anomalies in a video becomes a binary classification with the final output score in the range of [0, 1], where 0 (zero) means no anomaly detected, and 1 (one) means anomaly present. The architecture of the proposed framework is illustrated in Figure 2. It contains three principal parts: the first part is composed of three blocks, with each one including a 3D convolutional layer followed by a max-pooling layer, the stacking of convolutional layers aiming to capture both the temporal and the spatial features of the input video; the second part, is an LSTM architecture followed by a global max-pooling layer, which is used to further model the inter-related features from the first step and extract the most important ones for classification; and the third part, contains two dense layers to generate the final score of the video. 

#### 2.2.1. Convolutional Layers

As discussed in Section 2, the detection of anomalies in a video relies on the correct extraction of spatial and temporal features of the video. 3D convolutions have been proved to be effective in doing this task; thus, three convolution blocks are used. In the first layer of the first block, 4 filters are used, with a spatial kernel of 3×3 size, to find the spatial relationships on each video frame, and a temporal step of 2 to focus on the changes of objects and backgrounds between neighboring frames; the output after the convolution is then put into a max-pooling layer, but only spatial pooling is used to keep more information of sequential temporal features. 

The extracted features are used as the input for the second block where they are convoluted by 8 filters of 4×3×3 size. The temporal receptive field is increased to 4 so that the layer can capture features presented in a longer temporal duration. Meanwhile, the temporal pooling size in the following pooling layer is set as 2 to extract the more prominent features along with the time change. The increased temporal pooling is compensated by the increased number of filters in this block. 

In the last block, the number of filters is doubled again to 16, with the size of 8×3×3. The doubled temporal step aims for a further combination of information along with the temporal change, and more filters are used to compensate for the increased temporal pooling size (doubled to 4) to obtain the most prominent features. 

The output after these three blocks is a combination of the abstract spatial and temporal features ready to be used for further processing. Only three blocks are used here because 3D convolution is computationally expensive, especially when the spatial or temporal receptive field is large; also, too many max-pooling layers may suppress the contextual information too much, leading to the loss of the important temporal relationship between video frames. Consequently, features obtained in this step can still be called “local” features, and their long-temporal changes and relationship need to be further modeled. Fortunately, the convolution operations keep the temporal sequences of the extracted features, so these discrete spatial-temporal features can be processed as a time sequence series. The LSTM network is an efficient architecture to suit this requirement. 

#### 2.2.2. LSTM Architecture

**Figure 3:**
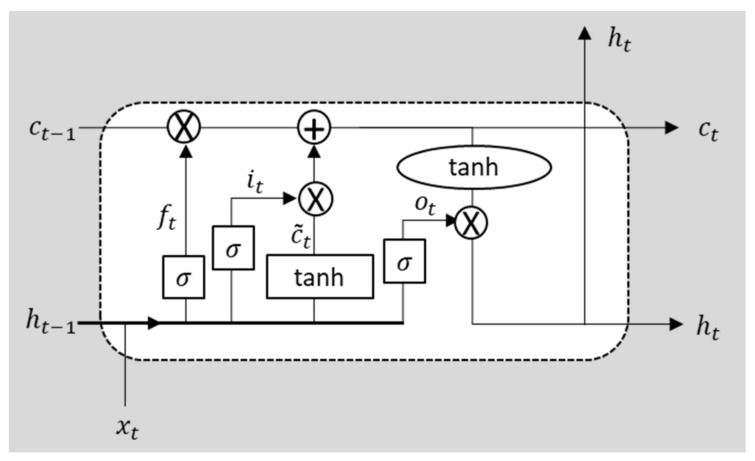
A memory unit in the LSTM architecture ($c_{t}$ is the cell state at the time step t, and $h_{t}$ is the hidden state used in the proposed method).

While the LSTM was initially developed to solve the vanishing gradient problem for training traditional RNNs, its insensitivity to gap length forms a big advantage over other sequence learning methods in many applications. A single memory unit in the LSTM architecture consists of a cell state and its three gates: an input gate, an output gate and a forget gate. Figure 3 illustrates the structure of a memory unit with the operations defined as follows:

(1) $$i_{t}=\sigma \left(w_{i}\left[h_{t-1},x_{t}\right]+b_{i}\right)$$

(2) $$f_{t}=\sigma \left(w_{f}\left[h_{t-1},x_{t}\right]+b_{f}\right)$$

(3) $$o_{t}=\sigma \left(w_{o}\left[h_{t-1},x_{t}\right]+b_{o}\right)$$

(4) $${\tilde{c}}_{t}=\tanh\left(w_{c}\left[h_{t-1},x_{t}\right]+b_{c}\right)$$

(5) $$c_{t}=f_{t}*c_{t-1}+i_{t}*{\tilde{c}}_{t}$$

(6) $$h_{t}=o_{t}*\tan h\left(c_{t}\right)$$

where $i_{t}$ is the input gate, $o_{t}$ is the output gate, $f_{t}$ is the forget gate, σ is the sigmoid function, tanh is the hyperbolic tangent function, $c_{t}$ and $h_{t}$ are the cell state and the hidden state of the time step t, w and b are the weights, operator ∗ stands for the element-wise product, respectively. Intuitively, the input information is selectively chosen, discarding the useless part and then combined with the prior information from its precedent unit through a series of nonlinear functions; the prior information is stored in the hidden state extracted from the previous inputs. Along with the training of LSTM, the hidden states store all the useful information from the previous sequences and can be seen as a substantial summary of those video frames, which is essential for the detection of anomalies.

Therefore, in the second part of the proposed framework, the features extracted in the first part are flattened according to their temporal sequence, and then the formed series is fed into the LSTM network for training. A total of 1024 units are used in the network to capture the memory features of each timestep along the timeline; therefore, along with the training of the LSTM network, the important spatial-temporal features for each time step are stored in the hidden state as a 1024-dimension vector. Given that videos are weakly classified, the state of the last cell of the LSTM network, which corresponds to the reminiscent information at the end of a video, may not contain any useful information for detecting anomalies that happened in the video (a case in point can be seen in Figure 1). Hence, instead of using only the states of the last cell of the LSTM network, all the hidden states are taken into account since they contain all the necessary information for detection. 

The hidden states are then put into a global max-pooling layer to extract the most prominent spatial-temporal features among different time phases. The output of the second part is a 1024-dimensional vector that represents the highly condensed contents of the video covering the abstract spatial-temporal relationships of the video frames and the most prominent features ready to be used for classification. Such a pooling operation in the hidden states aims to identify the anomalies even if they happen in a short time slot of a video; it does not require the video to be divided into a pre-defined number of segments and, therefore, is more flexible in finding anomalies when they are presented along with many normal scenes or events in a weakly supervised video. 

#### 2.2.3. Dense Layers

The third part of the framework is composed of two dense layers. The first layer contains 128 units and uses the rectified linear unit (ReLU) as the activation function:

(7) $$f\left(x\right)=\{\begin{array}{c} \;x\;\;\;\;\;\;\;\;\;\;\;\;\;\;if\;x>0\\ 0\;\;\;\;\;\;\;\;\;\;\;\;otherwise \end{array}$$

and the second layer, which is also the last layer of the framework, uses the sigmoid function to generate the final score. 

The final output of the proposed framework is a value ranging from 0 (zero) to 1 (one) to indicate whether the video contains anomalies; in the ideal case of binary classification, a score of 1 (one) means that a video contains anomalies, and a score of 0 (zero) means no anomaly in the video. Therefore, the following binary cross-entropy loss is used as the loss function:

(8) Loss=−tlog(p)−(1−t)log(1−p)

where t stands the ground truth score of the training sample and p stands for the output score of the framework. 

With the three-parts architecture and the loss function defined in Equation (8), the proposed framework can be trained by proper datasets and used for detection in new videos. The small kernel sizes of architecture facilitate the computation and implementation, which enables high efficiency with potential use for both offline and online detection.

## 3. Results

The proposed framework followed a sound procedure to extract the critical features for anomaly detection. To demonstrate its effectiveness and objectively compare with other state-of-the-art methods, experiments were carried out on two currently available weakly supervised datasets: UCF-Crime and XD-Violence datasets. 

The UCF-Crime dataset is a large-scale video dataset introduced in [5]. It consists of 1900 long and untrimmed real-world surveillance videos with a total duration of 128 h. The dataset has been further divided into 13 types of anomalies including abuse, arson, robbery, and road accident, and can be used for activity recognition. However, in our experiments, we discarded these further classifications and only considered a video as either normal or abnormal. As an objective comparison to the performance of other methods, the division of training and testing set follows the way defined in [5]: the training set contains 810 abnormal videos and 800 normal videos, while the testing set contains 140 abnormal videos and 150 normal videos. 

XD-Violence is another large-scale video dataset proposed for violence detection [7]. The dataset contains 4754 videos with a total duration of 217 h; besides the videos, audio signals were also provided so that multi-model fusion can be used to improve the detection accuracy. The videos were acquired from multi scenarios, including clips from games, movies, and YouTube. The training set is composed of 3953 videos; among them, 2048 videos contain no anomalies while the remaining 1905 ones contain different levels of violence. The dataset also provides sub-classifications according to the violence type; but, like in the UCF-Crime dataset, such information was discarded in the experiment. The testing set contains 300 normal videos and 500 abnormal videos. 

The two aforementioned datasets were selected because both are large-scale and contain a considerable number of training samples, so the framework is less likely to have problems of undertrained or overfitting.

### 3.1. Data Pre-Processing and Augmentation

The training of the proposed framework was straightforward and end-to-end. A video was treated as an integral input formed by sequential video frames and fed to the network. The aspect ratio of a video was assumed to be the traditional 4:3; so, for each video frame, the width and the height were rescaled to 160 and 120 pixels, respectively. All the color channels of the video frame were divided by 255 to normalize their values into the range [0, 1].

Given the training videos were weakly labeled, the exact spatial and temporal locations of the activities or objects, which determine the video attribute, are unknown. Consequently, some data augmentation techniques, like cropping and translation are not applicable due to their possible modification of sensitive information of an event or object. Nevertheless, a safe way was adopted to extend the training samples by generating a new training sample by flipping horizontally all the video frames; such a mirrored change does not lose any critical information for detection, nor alternates any critical features of activities when the background and context have the same alternation. Such augmentation of data can benefit the training, because it emphasizes the detection of events and activities themselves, without adding any artificial interpolation information to the process. 

### 3.2. Data Training

Following the procedure of LSTM, each video was fed into the framework as one integral input, so the batch number was set to 1 (one). The stochastic gradient descent (SGD) algorithm was used as the optimizer for both datasets with a learning rate of 0.0002. Hyper-parameters for each layer were fixed as introduced in Section 2 for both datasets. 

The proposed framework had no restriction on the number of frames contained in the input video. Therefore, the input videos can have any time duration and number of frames. However, if the time duration of a video was too long, the combined sequential video frames become a burden to the CPU/GPU memory and could lead to an overflow, either in the training when updating the weights, or in the testing when calculating the final score. To handle this problem, the maximum number of frames contained in an input video was set to be 4000; if a video contains more than 4000 frames, it was split into clips with each one containing 4000 frames (the last and the second last clip can have overlaps to satisfy this requirement). For each epoch of the training phase, a clip was randomly selected for the oversized video and fed into the network. For testing, each of the split clips was processed separately, and the final score of the video was defined as the maximum of these scores:

(9) $$S=\underset{i}{\max}S_{i}$$

where $S_{i}$ was the score of the i-th split video clip. This strategy was adopted only because of the consideration of computational limits from hardware.

## 4. Discussion

### 4.1. Quantitative Analysis

The proposed framework outputs a score ranging from 0 (zero) to 1 (one) that can be considered as the possibility of a video containing an anomaly. For such a binary classification, the area under the ROC curve (AUC) is a conventional index to show how accurate the classifier is. 

The receiver operating characteristic curve (ROC) is a graph showing the performance of a classifier at different thresholds; the vertical axis is the true positive rate (*TPR*), which is also called sensitivity, and the horizontal axis is the false positive rate (*FPR*). The calculations of *TPR* and *FPR* were defined as:

(10) $$TPR=\frac{TP}{TP+FN}$$

(11) $$FPR=\frac{FP}{FP+TN}$$

where *TP*, *FP*, *TN* and *FN* stand for the numbers of true positive, false positive, true negative, and false negative video samples in the testing set, respectively. AUC provides an aggregate index of performance across all possible thresholds for classification by measuring the area underneath the ROC curve from (0,0) to (1,1). The AUC shows how well the classification is without focusing on a specific threshold, indicating, therefore, the overall performance of a classifier. 

**Figure 4:**
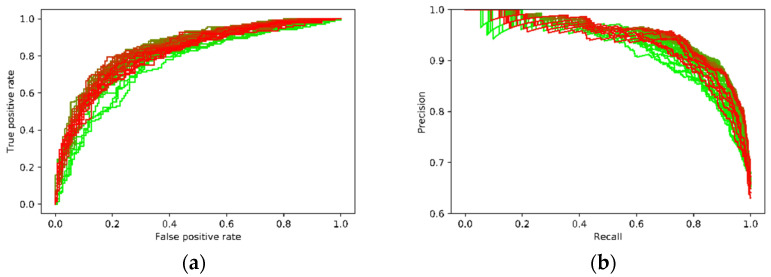
Performance on the two large-scale datasets: (**a**) The evolution of ROCs during the training for the UCF-Crime dataset (the color of the curve transits from green—early epochs, to red—late epochs); (**b**) the evolution of PRCs during the training for the XD-Violence dataset (the color of the curve transits from green—early epochs, to red—late epochs).

Given that the AUC is a commonly adopted index for the UCF-Crimes dataset, Figure 4a shows the changes of AUC for the 50 epochs of training; the highest AUC value is 0.8523. Table 1 listed the experimental results of the proposed framework along with the results of other state-of-the-art methods reported in the literature. To the best of our knowledge, it is currently among the best results concerning this dataset. 

For the XD-Violence dataset, the average precision (AP) of the precision-recall curve (PRC) was more frequently used to show the performance of a classifier. In a PRC curve, *TPR* becomes the horizontal axis, and the vertical axis is the precision calculated as:

(12) $$Precision=\frac{TP}{TP+FP}$$

where *TP* and *FP* follow the definition in Equations (10) and (11). The main difference between a ROC curve and a PRC curve is that the number of true negative samples is not used in PRC, so PRC focuses more on the positive cases, and is more used when there is class imbalance. Figure 4b illustrates the evolution of the PRC curves during the training, and Table 2 presents the performance of the proposed framework along with the ones reported by other existing methods. One can see that the proposed framework also achieved the highest AP score with an impressive enhance to 0.9517 from the baseline of 0.73 on this dataset. Results on both datasets demonstrated the effectiveness of the proposed framework.

### 4.2. Further Discussions

As can be realized from the data in Table 1 and Table 2, the proposed framework outperformed other state-of-the-art methods. It achieved a higher accuracy on the XD-Violence dataset than on the UCF-Crime dataset. One of the possible reasons was that the videos of the UCF-Crime dataset have lower resolutions because they were mainly acquired by surveillance cameras, while most of the videos in the XD-Violence were acquired by high-definition cameras and, therefore, have clearer representations of the objects and events ongoing. Also, the anomalies in the UCF-Crime dataset were acquired from different view angles, and sometimes in far-field view, which adds more difficulties to detection. 

Meanwhile, the XD-Violence dataset focuses on anomalies with violent activities, while the UCF-Crime dataset covers much more types of anomalies, so the large inter-variance among these types could decrease the detection performance. Also, though the UCF-Crime dataset contains a decent total number of videos with anomalies, the videos of each sub-categorical anomaly remain to be few. Hence, the trained framework can fail to detect certain types of anomalies, and the intra-variance inside the same type of anomaly may further decrease its performance. In summary, larger datasets that cover more samples and types of anomalies are still in demand to further improve detection accuracy.

**Figure 5:**
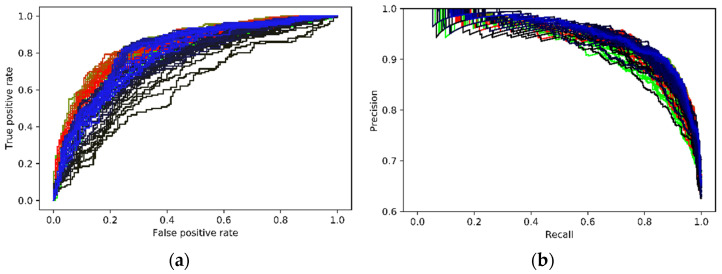
Performance on the fusion of the UCF-Crime and the XD-Violence datasets: (**a**) the evolution of AUCs; (**b**) the evolution of PRCs. (The curves were overlapped with the ones in Figure 4 to show the differences. The color of the curve transits from dark—early epochs, to blue—late epochs).

Given that the training of the new framework shared the same configurations for the two datasets, it would be interesting to see whether the performance of detection can be further improved if the two training datasets are merged. The results of combined training are illustrated in Figure 5. With more available samples and the diverse backgrounds of datasets, the training took longer time to achieve stable performance. One can see from the evolution of these curves that the combined training had a qualitatively better performance on the XD-Violence testing data (blue over red), but lower performance on the UCF-Crime testing data (blue under red). Quantitative analysis showed that the best AUC on the UCF-Crime dataset lowered to 0.8236, while the best AP on the XD-Violence dataset increased to 0.9528, slightly higher than the result obtained before. The two datasets have controversial tendencies of improvement. The combined training increased the true positive rate in the XD-Violence dataset without increasing too much false positive rate, which leads to a higher AP score. Nevertheless, since UCF-Crime covers more types of anomalies, one can see that compared to the training before, the false positive rate was higher, which means certain anomalies could not be detected without misclassifying normal videos, so the overall performance decreased. A possible reason for this phenomenon was that the normal videos in the training samples were more from the XD-Violence dataset than from the UCF-Crime dataset (2048:800), the imbalance may lead to different criteria on detecting whether a video is normal in the UCF-Crime testing data. Regardless, even with the lowered AUC score, the performance of the new proposed framework is still state-of-the-art.

## 5. Conclusions and Future Work

An efficient new framework was proposed to perform video anomaly detection. 3D convolutions and the LSTM architecture were used to extract the spatial and temporal features of videos for detection. The proposed framework followed the natural way of detecting anomalies in a video and has no restriction nor special pre-processing requirements on the video. The framework does not have restrictions on the temporal duration of the input video. It has a concise structure and is easy to be implemented; the efficient structure enables the possibility of both offline and online detection. Experiments on two large-scale weakly supervised datasets have been carried out, and the results demonstrated its effectiveness over other state-of-the-art methods. An exhaustive search in the hyperparameter space of the model may benefit even further the performance of the proposed method. Our future work will explore this aspect and devote to the fusion among multiple-channel information for detection; for example, by combining the videos with the sounds from the built-in microphone like the work carried out in [7] with the XD-Violence dataset.

Video anomaly detection has been a focus due to the large demands from different applications. The current framework achieved a state-of-the-art performance with a straightforward and effective architecture, but still has unsatisfactory performance in detecting certain types of anomalies. Further improvement and advances in video anomaly detection methods, rely on the availability of more large-scale video datasets that include sufficient training samples and cover various types of anomalies. To summarize, the main contributions of this study are:

- A new framework that provides an effective way of detecting anomalies by combining three-dimensional convolutions and the LSTM network.
- The structure of the new framework has high computational efficiency, which enables its application to videos with different resolutions and for different tasks.
- Experiments carried out in this study not only demonstrated the effectiveness of the new framework, but also improved the benchmarks on two large-scale datasets.

## Figures and Tables

**Table 1:** A comparison between the performances of the proposed framework and other state-of-the-art methods on the UCF-Crime dataset (best value found in bold).

Method	Main Features	AUC (%)
[5]	C3D [24]	75.41
[20]	C3D, TCN	78.66
[23]	TSN	82.12
[25]	C3D	83.03
[26]	I3D	82.30
[27]	C3D/I3D, RTFM	84.03
Our method	3D Convolution, LSTM	**85.23**

**Table 2:** A comparison between the performances of the proposed framework and other state-of-the-art methods on the XD-Violence dataset (best value found in bold).

Method	Main Features	AP (%)
[5]	C3D	73.20
[7]	C3D	67.19
[7]	C3D, Audio	78.64
Our method	3D Convolution, LSTM	**95.17**

## Data Availability

Not applicable.
